# *In vitro *and *in vivo *assessment of the anti-malarial activity of *Caesalpinia pluviosa*

**DOI:** 10.1186/1475-2875-10-112

**Published:** 2011-05-02

**Authors:** Ana Carolina AV Kayano, Stefanie CP Lopes, Fernanda G Bueno, Elaine C Cabral, Wanessa C Souza-Neiras, Lucy M Yamauchi, Mary A Foglio, Marcos N Eberlin, João Carlos P Mello, Fabio TM Costa

**Affiliations:** 1 Departamento de Genética, Evolução e Bioagentes, Instituto de Biologia, Universidade de Campinas (UNICAMP), Campinas, SP, Brazil; 2 Departamento de Farmácia Universidade Estadual de Maringá, Maringá, PR, Brazil; 3 Thomson Mass Spectrometry Laboratory, Instituto de Química, UNICAMP, Campinas, SP, Brazil; 4 Departamento de Microbiologia, Centro de Ciências Biológicas, Universidade Estadual de Londrina, Londrina, PR, Brazil; 5 Divisão de Fotoquímica, Centro Pluridisciplinar de Pesquisas Químicas, Biológicas e Agrícolas (CPQBA), UNICAMP, Campinas, SP, Brazil

## Abstract

**Background:**

To overcome the problem of increasing drug resistance, traditional medicines are an important source for potential new anti-malarials. *Caesalpinia pluviosa*, commonly named "sibipiruna", originates from Brazil and possess multiple therapeutic properties, including anti-malarial activity.

**Methods:**

Crude extract (CE) was obtained from stem bark by purification using different solvents, resulting in seven fractions. An MTT assay was performed to evaluate cytotoxicity in MCF-7 cells. The CE and its fractions were tested *in vitro *against chloroquine-sensitive (3D7) and -resistant (S20) strains of *Plasmodium falciparum* and *in vivo *in *Plasmodium chabaudi*-infected mice. *In vitro *interaction with artesunate and the active *C. pluviosa *fractions was assessed, and mass spectrometry analyses were conducted.

**Results:**

At non-toxic concentrations, the 100% ethanolic (F4) and 50% methanolic (F5) fractions possessed significant anti-malarial activity against both 3D7 and S20 strains. Drug interaction assays with artesunate showed a synergistic interaction with the F4. Four days of treatment with this fraction significantly inhibited parasitaemia in mice in a dose-dependent manner. Mass spectrometry analyses revealed the presence of an ion corresponding to *m/z *303.0450, suggesting the presence of quercetin. However, a second set of analyses, with a quercetin standard, showed distinct ions of *m/z *137 and 153.

**Conclusions:**

The findings show that the F4 fraction of *C. pluviosa *exhibits anti-malarial activity *in vitro *at non-toxic concentrations, which was potentiated in the presence of artesunate. Moreover, this anti-malarial activity was also sustained *in vivo *after treatment of infected mice. Finally, mass spectrometry analyses suggest that a new compound, most likely an isomer of quercetin, is responsible for the anti-malarial activity of the F4.

## Background

One of the principal reasons for malaria's high morbidity and mortality is the widespread presence of drug-resistant strains of the parasite, resulting in the dramatically decreased efficacy of the available anti-malarial drugs, such as chloroquine (CQ) and sulphadoxine-pyrimethamine (SP) [1].

The compounds most widely used to treat malaria, quinine and artemisinin, are derived from traditional medicine and plant extracts [2]. Quinine was the first drug successfully used to treat malaria. However, this alkaloid has a high level of toxicity and a short pharmacological half-life, which limit its use [2,3]. Currently, artemisinin-based combination treatment (ACT) is the therapy of choice for uncomplicated *Plasmodium falciparum *malaria in areas of widespread parasite CQ-resistance [4]. However, failure to clear parasites after ACT treatment has recently been reported on the Cambodia-Thailand border, and genes related to artemisinin resistance have been discovered [5-9]. Furthermore, no new class of anti-malarial has been introduced since 1996 [10], and the most successful malaria vaccine was only partially efficient and short lived [11]. Therefore, the discovery of new potential anti-malarial compounds is urgently needed.

*Caesalpinia pluviosa*, commonly named "sibipiruna", is a leguminous of the Fabaceae family that is originated from Brazil. This genus is a rich source of furanoditerpenoids and has demonstrated multiple therapeutic properties, including antiviral [12-14], antimicrobial [15,16], anti-inflammatory [17,18], and antioxidant [19,20] activities. Preliminary studies have shown that *C. pluviosa *crude extract (CE) had *in vitro *anti-malarial activity against a CQ-resistant strain [21]. However, that work focused only on the CE, and no parasite inhibition was observed in an *in vivo *test. In the present study, the *in vitro *anti-malarial activities of *C. pluviosa *extracts and the fractions effective against CQ- resistant and -sensitive *P. falciparum *strains, alone or in combination with artesunate, have been evaluated and identified.

The cytotoxic properties of these plant-derived materials were determined and the *in vivo *effects in *Plasmodium chabaudi*-infected mice were also studied. Finally, mass spectrometry analyses were carried out to characterize new potential molecules with anti-malarial activity.

## Methods

### Plant extraction and fractionation

**Figure 1:**
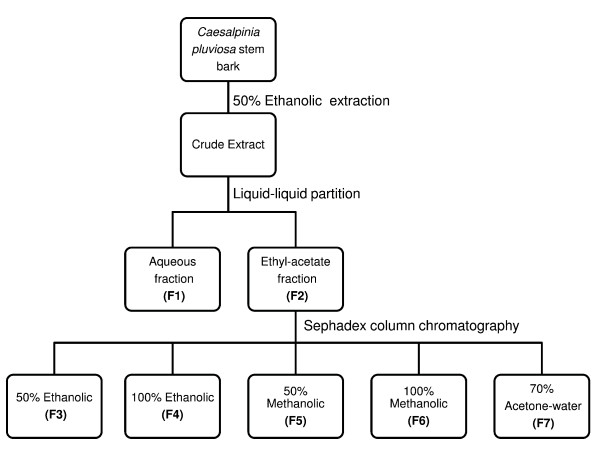
**Schematic view of the extraction and fractionation of *Caesalpinia pluviosa***.

Stem bark from *C. pluviosa *was collected and deposited at the campus of Universidade Estadual de Maringá, Brazil, in September 2006 as voucher #HUEM 12492. All plant material was ground and subjected to a turbo-extraction process with 50% ethanol-water for 15 min at a T<40°C. After evaporation of the organic solvent using a rotavapor under reduced pressure at 40°C, the CE (50 g) was lyophilized. CE was dissolved in water (500 mL) and extracted with ethyl acetate (10 × 500 mL). After removing the organic solvents by rotavapor under reduced pressure, aqueous (F1) and ethyl acetate (F2) fractions were formed. The F2 fraction was chromatographed by CC (chromatographic column) on Sephadex LH-20, resulting in five sub-fractions: 50% ethanolic-water (F3; 2.17 g), 100% ethanolic (F4; 0.21 g), 50% methanolic-water (F5; 0.06 g), 100% methanolic (F6; 0.17 g) and 70% acetone-water (F7; 0.08 g). All fractions were concentrated under reduced pressure at 40°C for solvent evaporation. These samples were lyophilized and used in biological tests, as described below. Concentrations of *C. pluviosa *CE/fractions were calculated on a dry material basis. Figure 1 summarizes the *C. pluviosa *extraction and its fractionation process.

### Cultivation of *Plasmodium falciparum *infected-erythrocytes *P. falciparum*

*Plasmodium falciparum *chloroquine-sensitive (3D7) [22] and chloroquine-resistant (S20) [23] strains were cultured in a candle jar as described elsewhere [24]. Briefly, *P. falciparum *infected-erythrocytes (Pf-iE) were cultivated in fresh-type O^+ ^human erythrocytes (UNICAMP, Blood Bank, Brazil) suspended at 4% final haematocrit in complete medium (RPMPI-1640 containing 10% homologous human plasma and 25 mM HEPES buffer, pH 7.4).

### Cytotoxicity assays

The cytotoxicities of the plant CE/fractions were assessed by MTT assay [3-(4,5-dimethylthiazol-2-yl)-2,5-diphenyl tetrazolium bromide] to evaluate their effects on the proliferation of a human breast cancer cell line, MCF-7 [25,26]. Cells were cultivated in DMEM/HAM-F12 medium supplemented with 10% heat-inactivated foetal bovine serum, penicillin (1 unit/mL) and streptomycin (1 unit/mL) in a humidified atmosphere of 5% CO_2 _at 37°C. MCF-7 cells were seeded in 96-well plates at a density of 2 × 10^4 ^cells per well and incubated with different concentrations of plant samples. After 48 h, 5 mg/mL of MTT solution was added for 4 h and formazan crystals were resuspended in 50 μL of isopropanol. The absorbances (A_590_) of cells containing medium (control) or in the presence of different concentrations of the CE/fractions (tested) were determined using an ELISA reader (Asys-Expertplus, UK). Data were calculated as the percentage of inhibition using the following formula: % inhibition = [1 - (A_t_/A_c_)] × 100. A_t _and A_c _refer to the absorbance of the tested *C. pluviosa *CE/fractions and the control, respectively.

The toxicity of the *C. pluviosa *CE/fractions was also determined in non-infected erythrocytes (niE) in the presence of different plant sample concentrations or untreated cells (control). The niE were cultured in complete medium at 37°C in candle jars, and the red blood cell density (RBCD) was determined after 48 h incubation with the aid of a Neubauer chamber. The percentage of RBCD, relative to 0 h, was calculated by the following formula: % RBCD = [1 - (n° niE treated with CE and fractions per hour after incubation/n° niE per or at 0 h)] × 100. Non-toxic samples were those in which no significant inhibition on MCF-7 growth or RBCD, relative to the control, was observed.

### *In vitro *anti-malarial activity of *C. pluviosa *extract and its fractions

[^3^H] hypoxanthine incorporation assays were used to determine *C. pluviosa *anti-malarial activity against Pf-iE growth as previously described [27]. Background values were determined by incubation with niE. Parasite growth in the presence of *C. pluviosa *extract and fractions (sample) was compared to control cultures (medium only). Inhibition of parasite growth was calculated according to the following formula: % inhibition = [1 - (n° niE treated with CE and fractions per hour after incubation/n° niE per or at 0 h)] × 100. The anti-malarial activities of the CE/fractions were classified according to their IC_50 _(μg/mL) values, defined as that concentration of compound which inhibits growth by 50% relative to untreated controls [21]. Fractions of IC_50 _< 5 were considered active, fractions of 10 > IC_50 _> 5 represented moderate activity and fractions with IC_50 _values > 10 were classified as inactive.

### *In vivo *anti-malarial activity of plant extract/fractions

*In vivo *anti-malarial activity of plant fractions was verified in C57BL/6 mice (7-10 weeks-old, weighing 20 ± 3 g) purchased from the Centro de Bioterismo-UNICAMP, Brazil, and maintained in specific pathogen-free animal facility. All experiments and procedures were approved by the UNICAMP Ethical Committee for Animal Research (protocol # 1806-1). Groups of 10 mice were infected intraperitoneally (i.p) with 10^6 ^iE of *P. chabaudi chabaudi *AS (PchAS). PchAs is a non-lethal strain kindly provided by Hernando Del Portillo (Department of Parasitology, ICB, USP, São Paulo-SP, Brazil and currently at CRESIB, Barcelona, Spain). One hour post-infection (p.i), groups of 10 animals were injected i.p with different concentrations (75, 50, 25 mg/kg/day) of the F4 and F5 fractions (50, 25 mg/kg/day) diluted in 200 μL PBS with 1.25% DMSO. Mice were treated for 4 days (day 0-3 p.i) according to previous studies [28,29]. Parasitaemia was monitored daily by microscopic examination of Giemsa-stained thin blood smears prepared from mouse tail blood beginning on the third day p.i. Mice in the control group received 200 μL of 1.25% DMSO diluted in PBS.

### *In vitro *compounds interaction on the Pf-iE

The artesunic acid (artesunate) used for combined treatment with F4 fractions was derived from artemisinin isolated from 1 kg of dried-plant material collected from CPQBA's experimental field (hybrid Ch × Viet 55) and extracted with ethanol as previously described [30]. Subsequent semi-synthesis procedures provided pure artesunic acid (98%), which was further dissolved in sodium bicarbonate solution (0.5%) prior to use as sodium artesunate on Pf-iE. Artesunate was identified by comparing the experimental product to a commercial sample (Aldrich^® ^98%, Sigma-Aldrich, USA). The 3D7 and S20 strains of *P. falciparum *(4% parasitaemia and 2% haematocrit) were incubated for 48 h in the presence of artesunate and the F4 fraction, solubilized in 1.25% DMSO. *C. pluviosa *fractions were dispensed into the 96-well micro-titre plates at different concentration (ng/mL) combinations of the F4 fraction and artesunate as follows: 4000-1.8, 2000-0.9, 1000-0.45, 500-0.225. The combination was performed by adding 50 μL of *C. pluviosa *fraction (4000 ng/mL) to 50 μL artesunate (1.8 ng/mL) and so on. Parasitaemia was analysed in thin blood smears, and the inhibition was compared to that of the control (100 μL of 1.25% DMSO) that represented 100% of Pf-iE growth. Corresponding IC_50 _values were determined for each drug alone and in combination [31]. The synergism degree was evaluated as described previously [32]. The sum of fractional inhibition concentration (SFIC) was calculated using the formula: K = A_c_/A_e _+ B_c_/B_e_, where K is the value corresponding to SFIC, A_c _and B_c _are the equally effective concentrations (IC_50_) when used in combination, and A_e _and B_e _are the equally effective concentrations used alone. The *in vitro *drug interaction was classified as follows: SFIC < 1 denotes synergism, 1 < SFIC < 2 denotes additive interaction, and SFIC≥2 denotes antagonism [33].

### Mass spectrometry

Samples of the F4 fraction were dissolved in HPLC grade MeOH, and 10 μL of this solution was diluted in 1 mL of solvent (MeOH/H_2_O [1:1] with 0.1% formic acid [v/v]). A Q-TOF mass spectrometer (Micromass, Manchester, UK) with an electrospray source was used to perform Electrospray Ionization/Mass Spectrometry (ESI-MS) and ESI-MS/MS analyses. The mass spectrometer was operated in the positive ion mode. The ESI source unit was operated at a desolvation temperature of 100°C, with a capillary voltage of 3.5 kV and cone voltage of 40 eV. Samples were directly infused at a rate of 10 μL min^-1 ^into the ion source using a syringe pump (Pump 11, Harvard Apparatus, Holliston, USA). The spectra were acquired in the interval of 100 to 2000 *m/z *and accumulated for 1 min. ESI-MS/MS were obtained for ions of interest using collision energies ranging from 10 to 50 eV. The collision gas pressure (argon) was optimized to produce extensive fragmentation of the ions under investigation. To calculate the theoretical masses of the compounds, MassLynx 4.1 software was used. The error among the theoretical and experimental masses was calculated according to the following formula: E = (m_ex _- m_t_/m_t_) × 10^6^; where m_ex _is an experimental mass and m_t _is a theoretical mass.

### Statistical analysis

Toxicity data were analysed using ANOVA or Mann-Whitney tests. Statistical significance between treated and non-treated *P. falciparum*-infected erythrocytes was determined using the Mann-Whitney *U *test. The inhibition of parasitaemia, resulting from sole or combined treatment with artesunate and F4, was compared using the Kruskal-Wallis test. For analysis of *in vivo *treatment with the F4 fraction, an ANOVA test was used. Calculations were performed using BioEstat™ version 3.0 (CNPq, Brazil), and values were considered significant when *p *< 0.05.

## Results

### Cytotoxicity assessment of *C. pluviosa*

The cytotoxic potential of the CE and fractions (F1-F7) was determined on MCF-7 cells by means of MTT assays. As shown in Table 1, plant CE from all fractions was highly cytotoxic at concentrations of 1600 and 400 μg/mL, significantly inhibiting cell growth from 20% (F4) to 66% (F6) (*p *< 0.05). In contrast, at 100 μg/mL the F4 and the F7 fractions did not significantly reduce cell growth compared to the control. The F1 and F5 fractions slightly, but significantly, inhibited MCF-7 cell development. No inhibition was observed by 25 μg/mL of any fraction. To determine if this low toxicity could be extant to niE, the RBCD percentage of plant CE and fractions relative to the control was determined at concentrations varying from 0.19 to 25 μg/mL.

**Table 1:** Growth inhibition (%)^a ^of MCF-7 cells *in vitro *treatment of *Caesalpinia pluviosa *assessed by MTT assay.

Concentrations (μg/mL)				
Samples	1600	400	100	25
CE	33.13 ± 10.88*	26.42 ± 19.26	21.95 ± 14.06	NT^a^
F1	45.43 ± 5.60*	44.21 ± 8.48*	6.98 ± 6.11	NT
F2	50.81 ± 13.25*	27.98 ± 7.15*	18.83 ± 17.15	NT
F3	41.67 ± 10.26*	44.31 ± 13.98*	45.73 ± 6.88*	NT
F4	55.90 ± 9.37*	20.19 ± 3.38*	NT	NT
F5	46.98 ± 11.73*	37.85 ± 7.86*	8.28 ± 2.56*	NT
F6	66.25 ± 5.45*	53.82 ± 4.69*	17.39 ± 14.27	NT
F7	62.66 ± 9.91*	44.93 ± 18.61*	NT	NT

^a^Values are expressed as the mean of triplicates ± SD.

^b^NT: Non toxic.

*p < 0.05

### *In vitro *determination of *C. pluviosa *anti-malarial activity

To evaluate the anti-malarial effects of plant CE/fractions against chloroquine-sensitive (3D7) and -resistant (S20) *P. falciparum *strains, parasite growth inhibition was measured by determining [^3^H] hypoxanthine incorporation at different concentrations (0.19 - 25 μg/mL). The anti-malarial activity IC_50 _values were compared for *C. pluviosa *CE and fractions. As shown in Table 2 and consistent with a previous study [21], the F1 and the F7 fractions were inactive (> 10 μg/mL) against both strains of parasites. However, the IC_50 _values of the CE and the other fractions were < 5 μg/mL, indicating inhibition activity. Of these, the F3 fraction and the CE presented higher IC_50 _values than the F4 and F5 fractions.

**Table 2:** *C. pluviosa *IC_50 _values (μg/mL) for crude extract and its fractions.

Plant Samples	IC_50 _3D7	IC_50 _S20
CE	4.84 ± 0.17	3.41 ± 2.45
F1	10.98 ± 6.01	13.29 ± 2.70
F2	2.13 ± 0.94	2.07 ± 1.38
F3	4.55 ± 2.05	5.49 ± 1.26
F4	0.72 ± 0.29	1.25 ± 0.38
F5	0.59 ± 0.33	1.72 ± 0.27
F6	1.30 ± 0.43	3.61 ± 2.46
F7	17.19	ND

The data shown are expressed as the mean of quadruplicates ± SD.

ND: Not determined.

**Figure 2:**
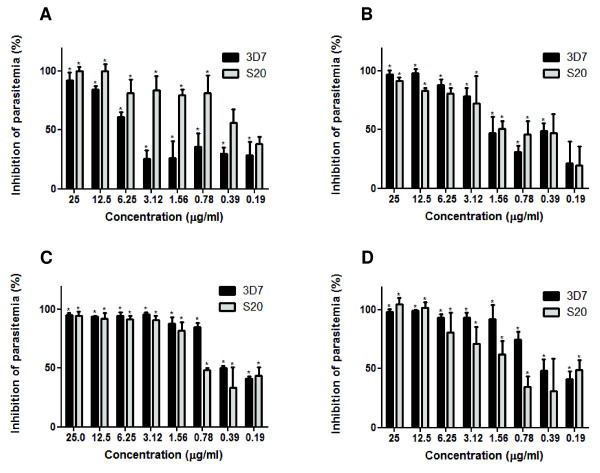
**Assessment of *C. pluviosa *anti-malarial activity against *P. falciparum***. Inhibition (%) of parasite growth of chloroquine-sensitive (3D7) and -resistant (S20) strains of *P. falciparum *cultivated 48 h at 37°C with *C. pluviosa *(**A**) crude extract and (**B**) ethyl acetate, (**C**) 100% ethanolic, (**D**) 50% methanolic fractions. The results are expressed as the mean of quadruplicates ± SD. (*p < 0.05 vs. untreated parasites).

The inhibition curve of the CE and the fractions that presented anti-malarial activity (IC_50 _< 5 μg/mL) are shown in Figure 2(A-D). The CE and the F2, F4 and F5 fractions were capable of inhibiting the growth of both parasite strains in a dose-dependent manner. The CE and all three fractions inhibited parasite development at concentrations of 25 and 12.5 μg/mL. At 6.25 μg/mL, inhibition was observed in the fractionated, but not crude extract. Notably, only the F4 fraction was able to sustain the inhibition on parasite growth throughout several concentrations, as shown in Figure 2C.

### Evaluation of the *in vivo *anti-malarial activity of *C. pluviosa*

**Figure 3:**
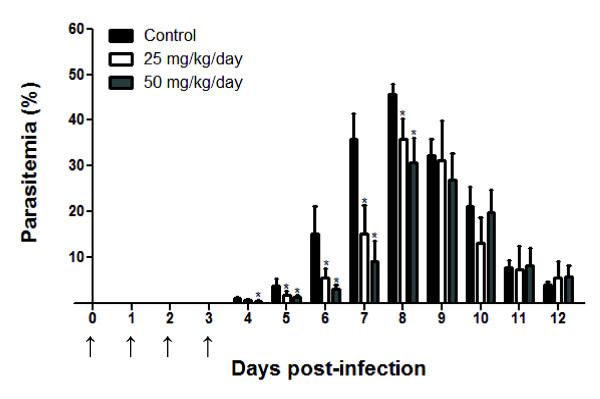
**Effect of the *C. pluviosa *F4 fraction on murine-derived *Plasmodium***. Groups of ten C57BL/6 mice infected i.p with 10^6 ^iE *Plasmodium chabaudi chabaudi *were left untreated or treated with different doses of the F4 fraction administered i.p for 4 consecutive days (days 0 to 3 p.i), starting on day 0 at 1 h p.i. The parasitaemia levels were determined daily until day 12 p.i. Results are expressed as the mean of a group mice ± SD. (*p < 0.05 vs. untreated mice group). The administration of F4 fraction is indicated by arrows.

After demonstrating the capacity of F4 and F5 fractions to control parasite burden *in vitro*, we evaluated whether this activity could be sustained in a mouse model of infection. *Plasmodium chabaudi*-infected mice were treated with different concentrations of both fractions for four days (day 0-3 p.i), starting at 1 h p.i. As shown in Figure 3, the parasitaemia of infected-mice was significantly reduced in a dose-dependent manner when the F4 fraction was administered at doses of 50 and 25 mg/kg/day during days 5-8 p.i. The highest dose (50 mg/kg/day) inhibited parasitaemia on days 6 and 7 p.i by 79.4% and 74.1%, respectively (Table 3). At the lower dose of 25 mg/kg/day, a significant reduction of parasite growth was also observed, although the percentage of inhibition achieved on day 6 p.i was 63.6% (Table 3). Doses of 75 mg/kg/day of the F4 and 50 mg/kg/day of the F5 were fatal to 40% of animals (n = 10) on days 2 and 4 p.i, respectively. In another set of experiments, 50 mg/kg/day of the F4 fraction (during days 3-7 p.i) inhibited parasite growth by 51% to 81% (Table 3).

**Table 3:** Parasitemia inhibition ^a ^of *Plasmodium chabaudi*-infected mice left untreated or treated with different doses of the 100% ethanolic fraction for 4 days (0-3 post-infection) from two independent experiments.

	Doses (mg/kg/day)	Days post-infection					
		D3	D4	D5	D6	D7	D8
Experiment							
# 1	50	ND	54.22 ± 14.20*	66.72 ± 13.16*	79.44 ± 5.77*	72.17 ± 12.28*	32.74 ± 11.59*
	25	ND	29.10 ± 11.51	51.46 ± 23.74*	63.64 ± 13.71*	57.45 ± 17.15*	21.50 ± 9.82*
# 2	50	81.16 ± 7.23*	90.45 ± 5.53*	91.06 ± 5.40*	86.04 ± 5.38*	51.04 ± 16.45*	NI

^a ^Values are expressed as the mean of parasitemia inhibition (%).

NI: No inhibition.

ND: Not determined.

* *p *< 0.05 vs. control

### Evaluation of the interaction between F4 fraction and artesunate

**Figure 4:**
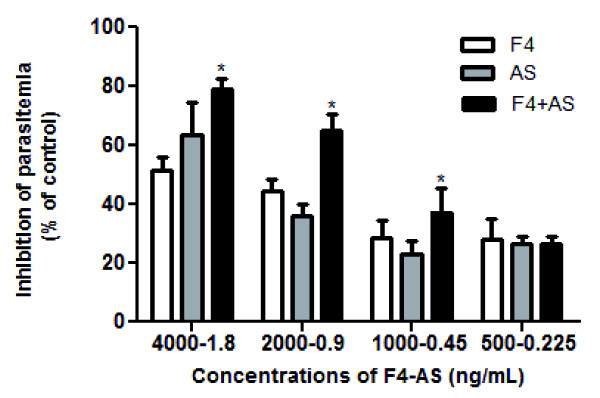
**Evaluation of the interaction between the F4 fraction and artesunate**. Inhibition (%) of parasite development (3D7) cultivated 48 h at 37°C with different concentration (ng/mL) combinations of the F4, artesunate (AS) or as a combined solution (F4+AS). The results are expressed as the mean of triplicates ± SD. (*p < 0.05 vs. non-combined treatment).

As shown in Figure 4, artesunate combined with the F4 fraction significantly reduced parasite development compared to the reduction observed when either compound was tested alone. The IC_50 _values (ng/mL) of the F4 fraction and artesunate alone were 3237 and 1.324, respectively, whereas the combination of the two led to values of 1402 and 0.630 respectively. Calculation of the SFIC value (0.908) indicated a synergic effect of the F4 fraction with artesunate.

### Molecular composition analysis of fractions from *C. pluviosa*

**Figure 5:**
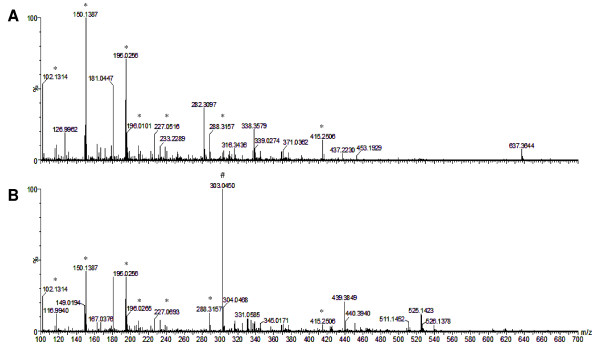
**Electrospray Ionization/Mass Spectrometry (ESI-MS) analyses of the (**A**) 70% acetone-water and (**B**) 100% ethanolic fractions**. Ions are marked either as those detected in both spectra (*) or only in the F4 fraction (#).

After assessing anti-malarial activity *in vitro *and *in vivo*, mass spectrometry was performed to characterize and identify the possible molecules involved in the anti-malarial activity. To accomplish this, the F4 (active) and F7 (inactive) fractions were ionized, and their spectra were analysed. As shown in Figure 5, their spectra displayed similarities, including *m/z *102.1314, 150.1387, 195.0256, 288.3157 and 415.2506. However, the most active fraction (F4) presented a distinct signal of *m/z *303.0450.

As these compounds were protonated [M+H]^+^, substances with a molecular mass (MM) of 302 in *Caesalpinia *spp. were searched, corresponding to the 303.0450. Table 4 shows all compounds found in the literature with 302 MM, which include ellagic acid [34], protosappanin C [35], sappanone B [36], 3'-deoxy-4-*O*-methylepisappanol [36], quercetin [37], and voucapen-5α-ol [38]. The high resolution and accuracy of mass spectrometry allows up to 20 portions per million (ppm) masses error. After calculating the theoretical masses of all retrieved compounds and the error among theoretical and experimental masses, quercetin was the only compound with an acceptable error (E = 18.15 ppm).

**Table 4:** Molecular composition identified in *Caesalpinia *spp.

Composition	Molecular formula	[M+1]^+^	E (ppm)	References
1	C_14_H_6_O_8_	303.0141	101.97	[34]
2	C_16_H_14_O_6_	303.0869	138.24	[35]
3	C_16_H_14_O_6_	303.0869	138.24	[36]
4	C_17_H_18_O_5_	303.1233	258.31	[36]
5	C_15_H_10_O_7_	303.0505	18.15	[37]
6	C_20_H_30_O_2_	303.2324	618.01	[38]

1. Ellagic acid, 2. Protosappanin C, 3. Sapponone B, 4. 3-deoxy-4-O- methylepisappanol, 5. Quercetin, 6. Voucapen-5α-ol. The compounds 2 and 3 are isomeric forms. Compounds with molar masses at 302 and its molecular formula were obtained from literature. [M+1]^+^, molar masses plus a proton. E: Error between theoretical and experimental masses.

**Figure 6:**
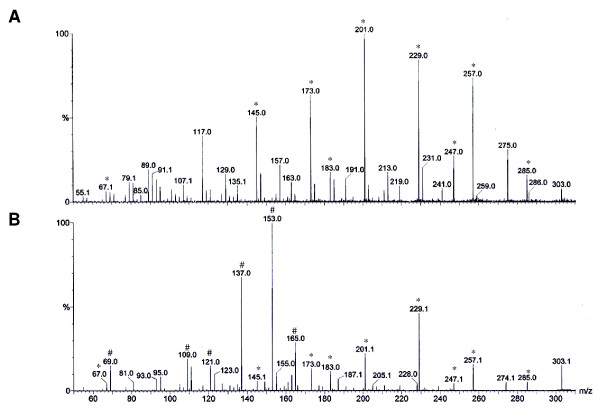
**ESI-MS/MS of the ion of *m/z *303 from the (**A**) 100% ethanolic fraction or from a (**B**) solution of quercetin standard**. Major fragment ions are marked as either detectable in both spectra (*) or only the spectra of the quercetin standard (#).

To confirm the hypothesis that quercetin was the corresponding molecule for the *m/z *303.0450 signal found in the F4 fraction, a new set of ESI-MS analyses using a quercetin standard (Sigma-Aldrich, USA) and the F4 fraction was performed (Figure 6). Although both compounds presented some similarities in their spectra, distinct signs of *m/z *137 and 153 were observed only in the quercetin standard.

## Discussion

Medicinal plants present a promising source of novel therapeutic agents for the treatment of many tropical diseases, including those caused by protozoa. These data confirmed the anti-malarial activity of the CE [21,39] and indicate that the F4 fraction possibly contains the main compound related to this activity.

When assessing anti-malarial activity *in vitro*, as classified previously [21], the CE/fractions of *C. pluviosa *have been presented inhibitory activity against chloroquine-sensitive (3D7) and -resistant (S20) strains of *P. falciparum*. These findings differ from previous studies [21,39], which reported that the CE is inactive against the chloroquine-sensitive strain (IC_50 _= 15 μg/mL). Moreover, the anti-malarial activity of the CE found here was twofold more efficient against the chloroquine-resistant strain (IC_50 _= 3.41 μg/mL) when compared with that found in the same previous studies (IC_50 _= 8.3 μg/mL). The discrepancy in IC_50 _values might be due to differences in the phytochemical and pharmacokinetic properties of the extract, which can vary depending on the origin, genotype and harvest period of the plants [40,41].

Because the F4 and F5 fractions presented the strongest inhibitory activity *in vitro*, these two fractions were chosen for evaluation in *P. chabaudi*-infected mice. After four days of treatment, our results showed that the anti-malarial activity of the F4 fraction was also very efficient *in vivo*. These data show that even after the fractionation and purification processes of the extract, no significant anti-malarial activity modification was observed. This is noteworthy because previous studies have reported that certain extracts and fractions have strong activity *in vitro*, but no inhibitory activity *in vivo *[42,43].

As ACT has been employed in areas with higher ratios of anti-malarial treatment failure, and interaction with artesunate has been a major step in drug discovery [44], the anti-malarial activity of the F4 fraction in the presence of artesunate was evaluated. Although few studies have shown *in vitro *synergistic interactions of natural compounds with artesunate [45], these analyses showed that artesunate was able to potentiate the reduction of Pf-iE development when combined with the F4 fraction, indicating that these distinct compounds had a synergistic effect. The fact that the interaction between F4 fraction and artesunate did not exhibit antagonistic interactions should prompt further exploration of novel therapeutic concentrations and combinations of other compounds from plants extracts for the treatment of malaria. As studies of drug combinations may reduce the risk of developing drug resistance and may lead to more effective therapeutic regimens for the treatment of malaria [44], a detailed evaluation of this synergic effect *in vivo *will certainly bring to light pertinent issues such as pharmacokinetics and pharmacodynamics of these compounds, solely or combined.

Mass spectrometry analyses of the F4 fraction detected an ion of *m/z *303.0450, similar to the fragmentation profile of quercetin (MM = 302), a natural flavonoid very common in edible fruits and vegetables [46]. Indeed, previous studies on the composition of *Caesalpinia *spp. have led to the isolation of several compounds, such as diterpenes [47-49], flavonoids [50], biflavonoids [51] and tannins [52]. Analyses of 480 plant-derived compounds have revealed that diterpenoids and flavonoids isolated from Caesalpiniaceae family are associated with anti-malarial activity against *P. falciparum *[53]. However, a second set of analyses using a quercetin standard showed distinct ions of *m/z *137 and 153. Furthermore, the IC_50 _value of the F4 fraction (0.72 μg/mL) obtained in these work was nine-fold lower than that of quercetin (6.5 μg/mL), as previously described [54], thus reinforcing the notion of a new compound related to the anti-malarial activity of F4 fraction, both *in vitro *and *in vivo *assays.

## Conclusion

The present study indicates that the F4 fraction of *C. pluviosa *has no cytotoxic effect and exhibits anti-malarial activity, both *in vitro *and *in vivo*. When combined with artesunate, this fraction potentiated the activity by significantly inhibiting parasitaemia. The presented findings suggest that a new compound, most likely an isomer of quercetin, is related to the anti-malarial activity of the F4 fraction.

## Competing interests

The authors declare that they have no competing interests.

## Authors' contributions

ACAVK carried out laboratory work, analysed the data and helped to draft the manuscript. SCPL contributed to the *in vitro *and *in vivo *anti-malarial activity assays. FGB and JCPM collected and fractionated the plant specimens. ECC and MNE participated in the mass spectrometry analysis and critically revised the manuscript. WCSN participated in the data analyses and helped to draft the manuscript, LMY helped in the design of experiments, and MAF participated in the experiments of drug combination. FTMC contributed to the study design and coordination, helped to interpret the data and drafted the final version of the manuscript. All authors read and approved the final manuscript.

## Acknowledgements and funding

Special thanks to André Batista Silva and André Spanhol for assistance in the extraction and purification process of plant extracts. This work received financial support from the Fundação de Amparo à Pesquisa do Estado de São Paulo (FAPESP), the Conselho Nacional de Desenvolvimento Científico e Tecnológico (CNPq), Instituto do Milênio de Desenvolvimento e Tecnologia em Vacinas, Instituto Nacional de Tecnologia em Vacinas (CNPq-FAPEMIG), CNPq-Doenças Negligenciadas (Grant no. 576128/2008-2) and CNPq-Universal (Grant no. 472913/2010-7). ACAVK was sponsored by FAPESP, and SCPL and WCSN received fellowships from CNPq and CAPES foundation (PNPD), respectively. FTMC is a CNPq fellow. The sponsors had no role in study design, data collection and analysis, the decision to publish, or preparation of the manuscript.
